# Integrated RNA-seq and snRNA-seq analysis identifies *PR10* tandem gene cluster governing early defense against *Fusarium wilt* in sea island cotton

**DOI:** 10.3389/fpls.2025.1622223

**Published:** 2025-11-05

**Authors:** Baojun Liu, Shu Wang, Yujia Zhang, Gang Liu, Ziyi Liu, Aixing Gu, Jianyu Bai

**Affiliations:** ^1^ College of Agriculture, Xinjiang Agricultural University, Urumqi, Xinjiang, China; ^2^ Engineering Research Centre of Cotton, Ministry of Education, Urumqi, Xinjiang, China; ^3^ Institute of Forest Ecology and Landscaping, Xinjiang Academy of Forestry Sciences, Urumqi, Xinjiang, China

**Keywords:** *Gossypium barbadense*, FOV7, transcriptome, PR10, tandem duplication, single-nucleus RNA sequencing

## Abstract

*Fusarium* wilt, caused by *Fusarium oxysporum f.* sp. *vasinfectum 7* (FOV7), poses a major threat to the production of elite Sea Island cotton (*Gossypium barbadense*). To uncover the molecular basis of defense FOV7 in cotton, we employed RNA sequencing to identify numerous differentially expressed genes across various stages of infection. Subsequent K-means clustering and weighted gene co-expression network analysis revealed a core module significantly enriched in defense response and abscisic acid-activated signaling pathways. A detailed examination of the gene distribution within these pathways identified 10 out of 50 genes as members of the *Pathogenesis-Related 10* (*PR10*) gene family. Evolutionary analysis of these *PR10* genes uncovered a tandemly-expanded gene cluster located on chromosome 10 of the D sub-genome. In addition, root cell type maps constructed via single-nucleus RNA sequencing (snRNA-seq) enabled pinpointing FOV7 response in the root epidermis, where *GbD_PR10.11* was identified as a specifically activated sentinel. Our work, by logically progressing from genome-wide patterns to a single gene in a single cell type, not only deciphers a key component of the cotton-pathogen arms race but also delivers a high-confidence target for engineering frontline resistance.

## 1 Introduction

Sea Island cotton (*Gossypium barbadense L.*), renowned for producing the world’s most valuable long-staple fibers, is a crop of immense economic significance (Rehman et al., 2019). However, its production is severely threatened by Fusarium wilt, a devastating soil-borne disease caused by *Fusarium oxysporum f.* sp. *vasinfectum Race* (FOV) (Bell et al., 2019; Michielse and Rep, 2009; Sanogo and Zhang, 2015). As a vascular wilt pathogen, FOV7 invades the root system, colonizes the xylem vessels, and systemically spreads throughout the plant. This colonization obstructs water transport, leading to characteristic symptoms of stunting, wilting, vascular browning, and ultimately, plant death, resulting in substantial economic losses (Dastmalchi et al., 2019; Diaz et al., 2021). Despite progress in identifying quantitative trait loci and defense-related genes, a fundamental aspect of the host-pathogen interaction remains limited: the initial molecular events occurring at the cellular level in the root, where the infection begins (Abdelraheem et al., 2024; Liu et al., 2021; Zhang et al., 2022).

Modern genomic technologies have provided valuable resource of the transcriptome-wide response to FOV infection (Ning et al., 2021; Ojeda-Rivera et al., 2023; Prasath et al., 2023). Transcriptomics technologies have been extensively utilized in investigating the defense mechanisms of cotton under stress conditions, offering novel insights that significantly enhance our understanding of cotton’s survival and adaptive strategies in response to diverse stresses (Khan et al., 2023; Luqman et al., 2025). These studies have successfully identified numerous defense-related genes, yet they suffer from an inherent limitation: they measure the average expression across a heterogeneous mix of root cell types. This averaging effect makes it impossible to distinguish the unique responses of different cellular type (Tang et al., 2023). Recent advances in single-cell transcriptomics have begun to resolve such complexity in other plant-stress contexts, such as mapping the salt-stress response in diploid cotton roots (Li et al., 2024) and dissecting cell-specific immunity in *Arabidopsis* leaves infected by a fungal pathogen (Tang et al., 2023). However, a cellular-level atlas of the pathogen response in the roots of tetraploid cotton has been missing.

Among the diverse arsenal of plant defense proteins, the Pathogenesis-Related 10 (PR10) family emerges as a prime candidate for orchestrating these rapid, localized responses (Chen et al., 2024). These small, acidic intracellular proteins are defined by a conserved Bet_v_1 domain, which confers a unique three-dimensional structure capable of binding various ligands and, in many cases, exhibiting ribonuclease (RNase) activity to directly inhibit pathogen growth (Lebel et al., 2010). The role of *PR10* has been explored in several crops, each showing different responses to various pathogens. In soybean, the *PR10* gene is induced during infection with *Phytophthora sojae* (Xu et al., 2014). In grapes, the *PR10* gene exhibits specific subcellular localization and function in response to *Plasmopara viticola* infection (He et al., 2013). In roses, the PR10 protein (*RC4G0290000*) inhibits pathogen expansion through ribonuclease activity after grey mold infection and coordinates defense responses through subcellular localization regulation (Li et al., 2024). In rice, the expression of the *OsPR10* gene is regulated by jasmonic acid and ethylene signaling pathways, while salicylic acid inhibits its expression (Yamamoto et al., 2018). Additionally, resistance genes in cabbage indirectly enhance *PR10*-mediated systemic resistance by recruiting beneficial rhizosphere microorganisms to regulate the expression of ethylene and JA pathway genes (Ping et al., 2024). In cotton, *GbPR10-5D1* can surprisingly increase susceptibility to *Verticillium dahliae* (Guo et al., 2022). Despite extensive studies in other species, the specific roles and cellular deployment of the PR10 family within cotton roots during the initial confrontation with FOV7 are entirely unknown.

In this study, we aimed to dissect the cellular and molecular architecture of cotton’s early defense against FOV7. We hypothesized that an integrated approach, combining the breadth of bulk RNA-seq with the precision of single-nucleus RNA-seq (snRNA-seq), could resolve this complex picture. Our multi-scale analysis first identified a key module of rapidly induced *PR10* genes through transcriptomic profiling. We then revealed that a tandemly duplicated gene cluster on chromosome D10 is a genomic hotspot for this response, suggesting an evolutionary adaptation for a robust defense. Finally, by generating a single-nucleus atlas of the infected root, we pinpointed the epidermis as a key defensive battleground and identified *GbD_PR10.11* as a sentinel gene specifically activated in these frontline cells. This work provides an unprecedented high-resolution view of cotton’s early immunity and offers precise targets for future resistance breeding.

## 2 Materials and methods

### 2.1 Plant materials, growth conditions, and pathogen inoculation

The *Gossypium* barbadense cultivar “Xinhai 14” was used throughout this study. This cultivar was selected as it is a major, elite commercial variety of Sea Island cotton and is known to be susceptible to FOV7 (Han et al., 2022; Zu et al., 2019), thus providing a relevant and suitable model for investigating the molecular mechanisms of the early host-pathogen interaction. The seeds were surface-sterilized with 0.1% HgCl_2_ and then placed in a constant-temperature germination chamber (28 ± 0.5°C) for germination. When the primary root of the seedlings reached a length of 3 cm, the seedlings were transplanted into a hydroponic system and cultured using Hoagland’s solution (pH 6.0 ± 0.2). The culture conditions were set as follows: a photoperiod of 16 h light/8 h dark, a light intensity of 100 μmol·m>⁻²·s⁻¹ (using full-spectrum LED light), a relative humidity of 65 ± 5%, and a constant temperature of 28 ± 1°C.

The *Fusarium oxysporum f.* sp. *vasinfectum race 7* (FOV7) strain was cultured on PDA medium at 25°C for 5 days, followed by culture in liquid PDB medium for 7–10 days at 25°C on a shaker (180 rpm). The final spore concentration was adjusted to 1×10^8^ spores/mL with 0.01% Tween-20 as a surfactant, and the suspension was used immediately for inoculation to ensure maximal viability.

When the seedlings had fully expanded their second true leaves (14 days after sowing), the pathogen was inoculated using the root-drench method, with an inoculum volume of 5 mL per plant. Root tip tissue samples were collected at four key time points (0 hpi, 2 hpi, 12 hpi, and 48 hpi) after inoculation. Immediately after sampling, the samples were flash-frozen in liquid nitrogen and stored in a -80°C ultra-low-temperature freezer for later use.

### 2.2 Bulk RNA-seq library preparation and data analysis

We followed the standard RNA-seq library construction protocol, selecting four key time points (0 hpi, 2 hpi, 12 hpi, and 48 hpi), with three biological replicates for each time point (Chen et al., 2024). Qualified libraries were constructed and sequenced on an Illumina NovaSeq X Plus platform (PE150). After quality control, clean reads were aligned to the *G. barbadense* reference genome (H7124_ZJU, downloaded from the CottonMD database) (Yang et al., 2023) using HISAT2 (v2.2.1) (Kim et al., 2015). Gene expression was quantified as FPKM using StringTie (v2.2.0) (Pertea et al., 2015). DEGs were identified using DESeq2 (v1.42.0) with thresholds of |log_2_ fold change| > 1 and FDR < 0.05 (Love et al., 2014). These widely-used and stringent thresholds ensure the identification of genes with both statistically significant and biologically meaningful changes in expression (Love et al., 2014). GO and KEGG pathway enrichment analyses were performed using the clusterProfiler R package (v4.10.1) (Wu et al., 2021).

### 2.3 Expression pattern clustering and WGCNA

K-means clustering was performed on all DEGs using the ClusterGVis R package (v0.1.2) (Kumar and E Futschik, 2007). WGCNA was conducted using the WGCNA R package (v1.71) (Langfelder and Horvath, 2008) with a soft-thresholding power of 10, a minimum module size of 30, and a merge cut height of 0.20.

### 2.4 Bioinformatic analysis of the *PR10* gene family

To identify the *PR10* gene family, the genome sequence and annotation data of *Gossypium barbadense* and *Gossypium arboreum* (A2 genome) were obtained from CottonMD (https://yanglab.hzau.edu.cn/CottonMD/), *Gossypium raimondii* (D5 genome) were downloaded from NGDC (https://ngdc.cncb.ac.cn/gwh/Assembly/84056/show), and the *Arabidopsis thaliana* from TAIR (https://www.arabidopsis.org/). *PR10* genes were initially screened in the genome using HMMER 3.0 (Finn et al., 2011). The search was guided by the hidden Markov model (HMM) corresponding to the Bet_v_1 domain (PF000407), which was obtained from the PFAM database. A candidate *PR10* gene was defined as one that contained the Bet_v_1 domain, with an e-value threshold set to < 1e-10. To ensure accuracy, the conserved domains of these candidate *PR10* genes were further validated using the SMART database (https://smart.embl.de/) and the NCBI-CDD platform (https://www.ncbi.nlm.nih.gov/Structure/cdd/cdd.shtml). The *PR10* gene families in other species were identified using the same method.

To elucidate the evolutionary relationships of PR10 proteins, the complete PR10 protein sequences from the four species were aligned using MAFFT v7.4.1 (Katoh and Toh, 2008). An unrooted phylogenetic tree was constructed by the maximum likelihood (ML) method in MEGA 11 (Kumar et al., 2018), with statistical support assessed via 1,000 bootstrap replicates. The resulting phylogenetic tree was subsequently visualized using Evolview v3 (Subramanian et al., 2019).

The chromosomal positions of *PR10* genes were determined from the annotation files of the three cotton genomes and visualized using TBtools-II (Chen et al., 2023). Gene synteny analysis was performed using MCScanX (Wang et al., 2012), and gene duplication events were classified using the duplicate_gene_classifier tool. Furthermore, Ka and Ks values for the *PR10* genes were calculated with KaKs_Calculator 3.0 (Wang et al., 2010), and the Ka/Ks ratio was computed to evaluate the selection pressure acting on these genes.

### 2.5 Single-nucleus RNA-seq library preparation and analysis

We utilized the same samples from bulk RNA-seq to conduct single-cell RNA sequencing using normal cotton root tips and those inoculated by FOV7 at 2 hours. A high-quality single-cell suspension was successfully prepared and carefully loaded onto the MobiNova-100 high-throughput single-cell controller (MobiDrop, Zhejiang, China). The single-cell RNA sequencing library was meticulously constructed using the MobiCube RNA-seq single-cell kit (MobiDrop, Zhejiang, China). Subsequently, these libraries were sequenced on the MobiNova-100 single-cell sequencing platform (MobiDrop, Zhejiang, China) using the Illumina NovaSeq 6000 sequencing strategy. To ensure the reliability and reproducibility of the data, each sample was sequenced in duplicate biological replicates.

We first removed low-quality reads, poly-A tails, and adapter sequences and obtained clean reads. Subsequently, we processed the clean data using the professional Mobivision software (https://www.mobidrop.com/bioinformatics/mobivision2) to construct an accurate expression matrix and then employed the Seurat package 4.4.0 (Hao et al., 2021)for downstream analysis. To ensure high-quality data, we applied stringent filtering criteria. Specifically, cells with fewer than 500 or more than 6,000 UMI counts were discarded, and genes detected in fewer than three cells were excluded. Additionally, only cells with less than 10% mitochondrial gene alignment transcripts were retained for further analysis. In the DoubletFinder (McGinnis et al., 2019)workflow, we retained only the cells annotated as “Singlets” in each library to ensure the accuracy of the single-cell data.

For data integration, we used the CCA (Canonical Correlation Analysis) algorithm (Hao et al., 2021). Dimensionality reduction was performed with the RunUMAP function, and clustering analysis was conducted with the RunPCA function using npc=30. We then accurately identified cell clusters using the “FindNeighbors” function (with parameters k.param=10 and dims=1:30) and the “FindClusters” function (with a resolution of 0.6). To identify DEGs in each cluster, we employed the “FindAllMarkers” function with the Wilcoxon rank-sum test. This allowed us to define DEGs between each cluster and all other cells. We annotated cell types using the PCMDB database (Jin et al., 2022). We used the “FindMarkers” function with parameters logfc.threshold = 1, min.pct = 0.25, and min.diff.pct = 0.1 for differential expression analysis between normal and FOV7 infection samples.

### 2.6 Real-time quantitative PCR validation

Total RNA was extracted and the integrity of the nucleic acids was confirmed by agarose gel electrophoresis, while the concentration and purity were assessed using ND5000 NanoDrop UV-Vis Spectrophotometer (Thermo Fisher Scientific Inc., USA). cDNA was synthesized from 200 ng of RNA using a reverse transcription kit (Hifair™ III 1st Strand cDNA Synthesis SuperMix for qPCR, YEASEN). The primers were designed using Primer 5.0s and are listed in **Supplementary Table S16**. Following the standard protocol, the Quantitative PCR was performed using on Bioer Line Gene 9600 Plus Real Time Thermalcycler (FQD-96A, Hangzhou Bori Technology Co., Ltd). Relative expression levels were calculated using the 2-ΔΔCT method (Rao et al., 2013) using the housekeeping gene *GAPDH*, and the data were visualized using GraphPad Prism version 10.1.2 (https://www.graphpad.com). The experiment included three technical replicates and three biological replicates.

## 3 Results

### 3.1 Transcriptome profiling reveals a rapid and robust early defense response to FOV7

**Figure 1:**
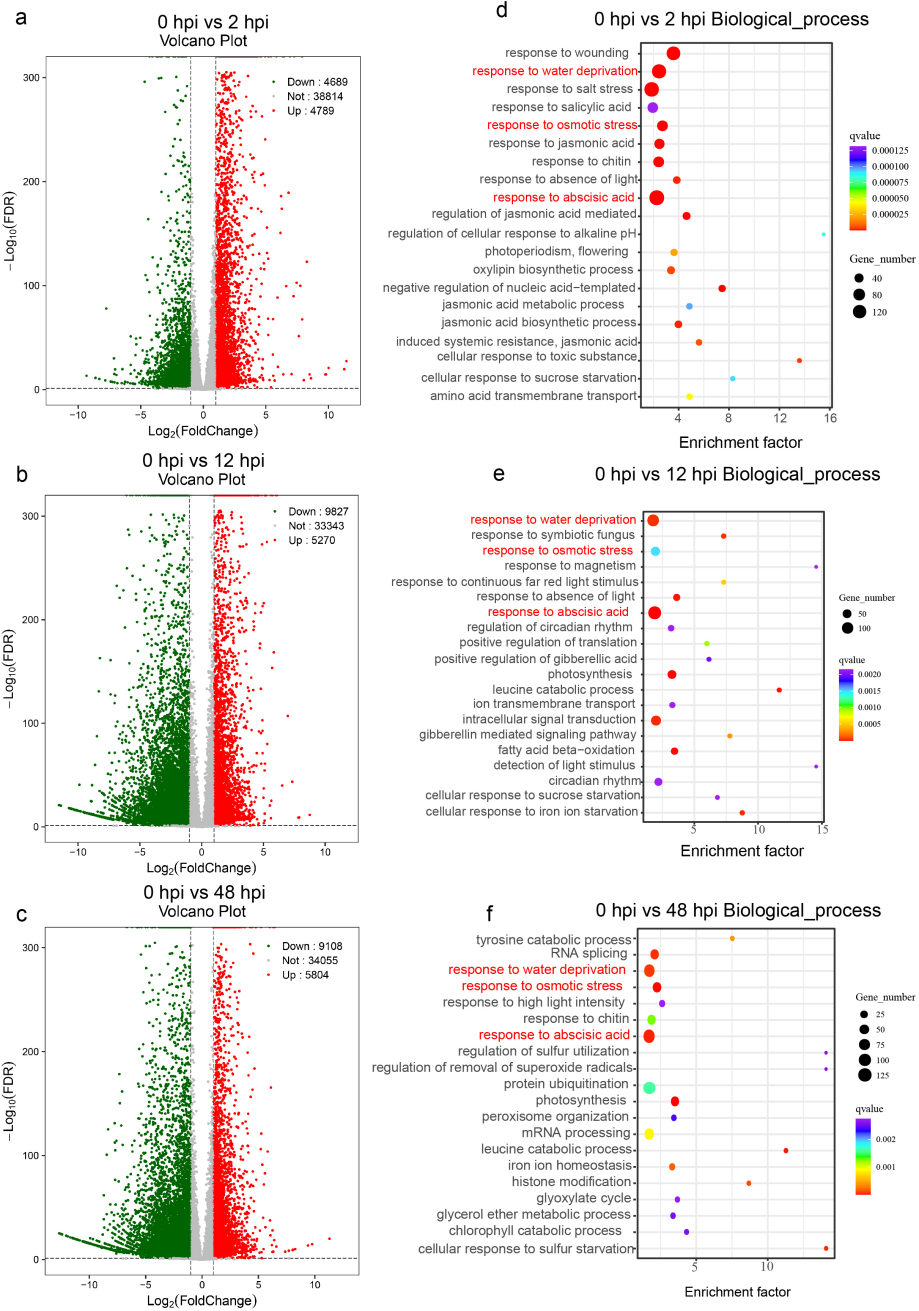
Differential expression analysis of the cotton root at various time points post-FOV7 infection. The volcano plots illustrate the differentially expressed genes of the “XinHai14” cultivar between uninfected roots (0 hpi) and roots infected with FOV7 at 2 hours **(a)**, 12 hours **(b)**, and 48 hours **(c)**. The top 20 Gene Ontology biological process enrichment analyses for upregulated genes are shown at 2 hours **(d)**, 12 hours **(e)**, and 48 hours **(f)**. The pathways highlighted in red in **(d–f)** represent the shared pathways.

To capture the temporal dynamics of the defense response in *G. barbadense* roots following FOV7 infection, we performed bulk RNA-seq at 0, 2, 12, and 48 hpi. We generated a total of 286,236,783 high-quality reads with an average mapping rate of 89.84% to the reference genome (**Supplementary Table S1**). Differential expression analysis revealed a massive transcriptional reprogramming. At the early infection stage of 2 hpi, we identified 9,478 DEGs, comprising 4,789 upregulated and 4,689 downregulated genes, indicating a swift and extensive cellular reaction (**Figure 1a**; **Supplementary Table S2**). The number of DEGs remained high at 12 hpi (5,270 up, 9,837 down) and 48 hpi (5,804 up, 9,108 down) (**Figures 1b, c**; **Supplementary Tables S3**, **S4**). GO enrichment analysis of the upregulated genes at all time points consistently highlighted multiple stress-related biological processes, including response to abscisic acid (GO:0009737), response to osmotic stress (GO:0006970), and response to water deprivation (GO:0009414), indicating a sustained and multifaceted defense activation (**Figures 1d–f**; **Supplementary Figure S1**).

### 3.2 Convergent analyses pinpoint a key *PR10* gene module in the early response

**Figure 2:**
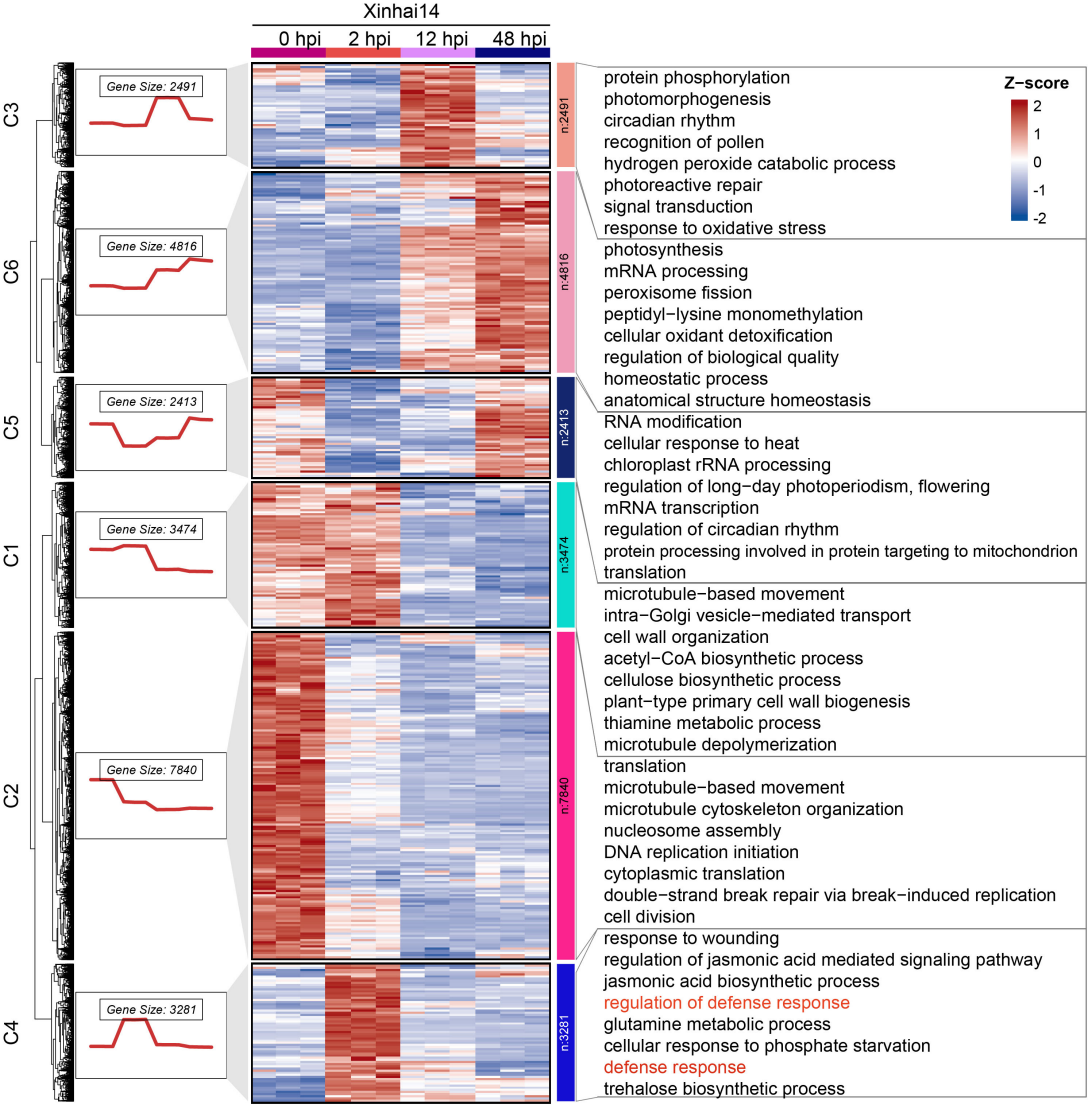
K-means clustering analysis and GO enrichment. On the left side was the gene expression trend charts for each cluster, with the numbers above indicating the number of genes in that cluster. In the middle is the heatmap corresponding to the normalized expression levels. On the right are the GO Biological Process (BP) enrichment analysis charts for each cluster, showing only the top 8 most significant terms.

To identify genes involved in the critical initial defense phase, we first employed K-means clustering on all DEGs, yielding six distinct expression clusters (**Figure 2**; **Supplementary Table S5**). Notably, genes in Cluster C4 exhibited a sharp and significant upregulation specifically at 2 hpi, followed by a gradual decline. GO analysis of this cluster revealed strong enrichment in terms like defense response (GO:0006952) and response to wounding (GO:0009611), suggesting its central role in the immediate immune reaction (**Figure 2**; **Supplementary Table S6**).

**Figure 3:**
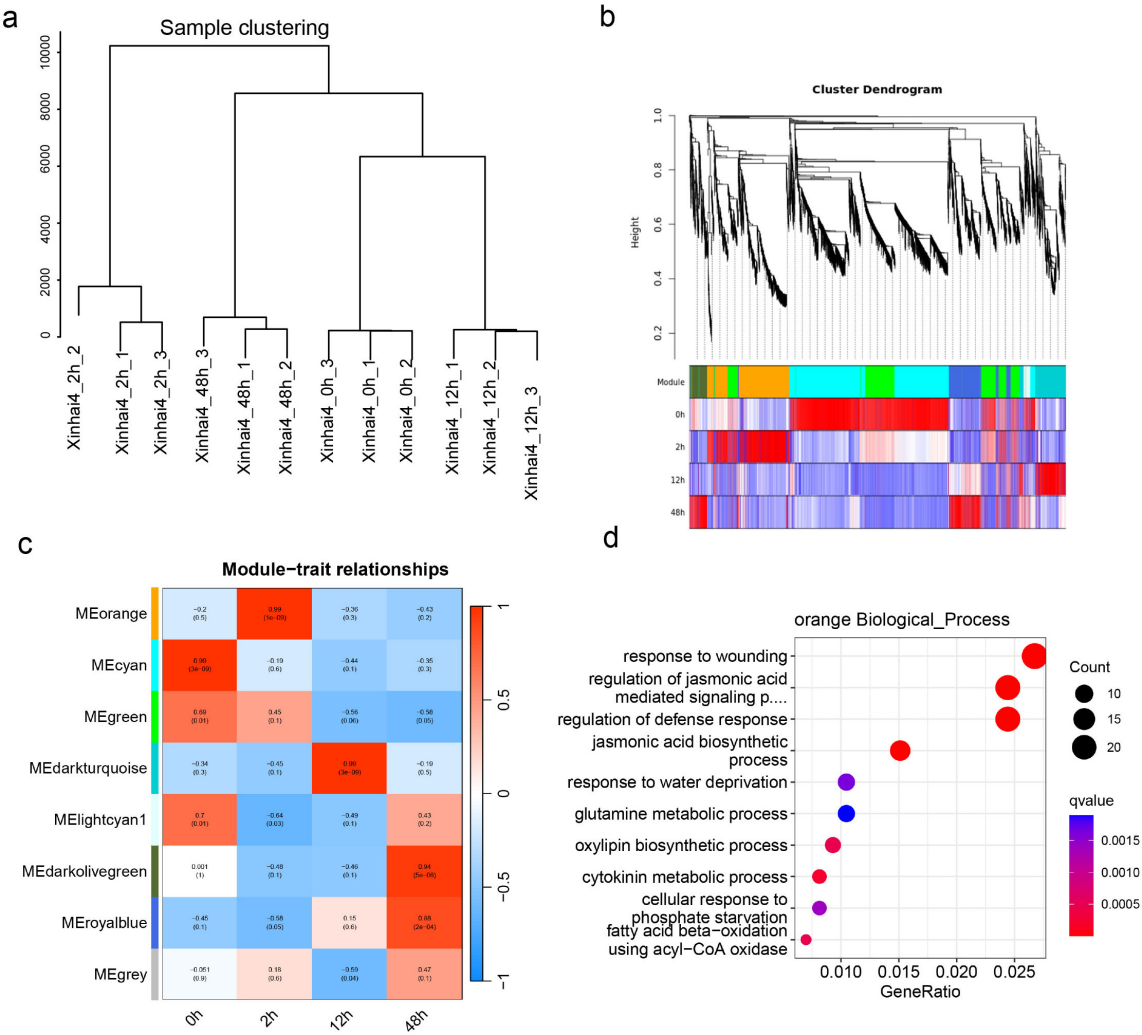
WGCNA analysis of cotton root responses to FOV7 Infection. **(a)** Sample clustering. **(b)** Cluster dendrogram of 20,163 DEGs with assigned module colors. **(c)** Heatmap of module-trait associations. **(d)** Gene Ontology biological process enrichment analysis of genes in the orange module.

To further refine this finding from a network perspective, we conducted Weighted Gene Co-expression Network Analysis (WGCNA), which grouped the DEGs into eight co-expression modules (**Figures 3a, b**). Correlating these modules with the infection time points, we identified the orange module (1,701 genes) as being highly and positively correlated with the 2 hpi stage (**Figure 3c**). GO enrichment of the orange module genes confirmed their involvement in processes such as response to wounding (GO:0009611), regulation jasmonic acid biosynthetic process (GO:2000022), and regulation of defense response (GO:2000022) (**Figure 3d**; **Supplementary Table S7**).

**Figure 4:**
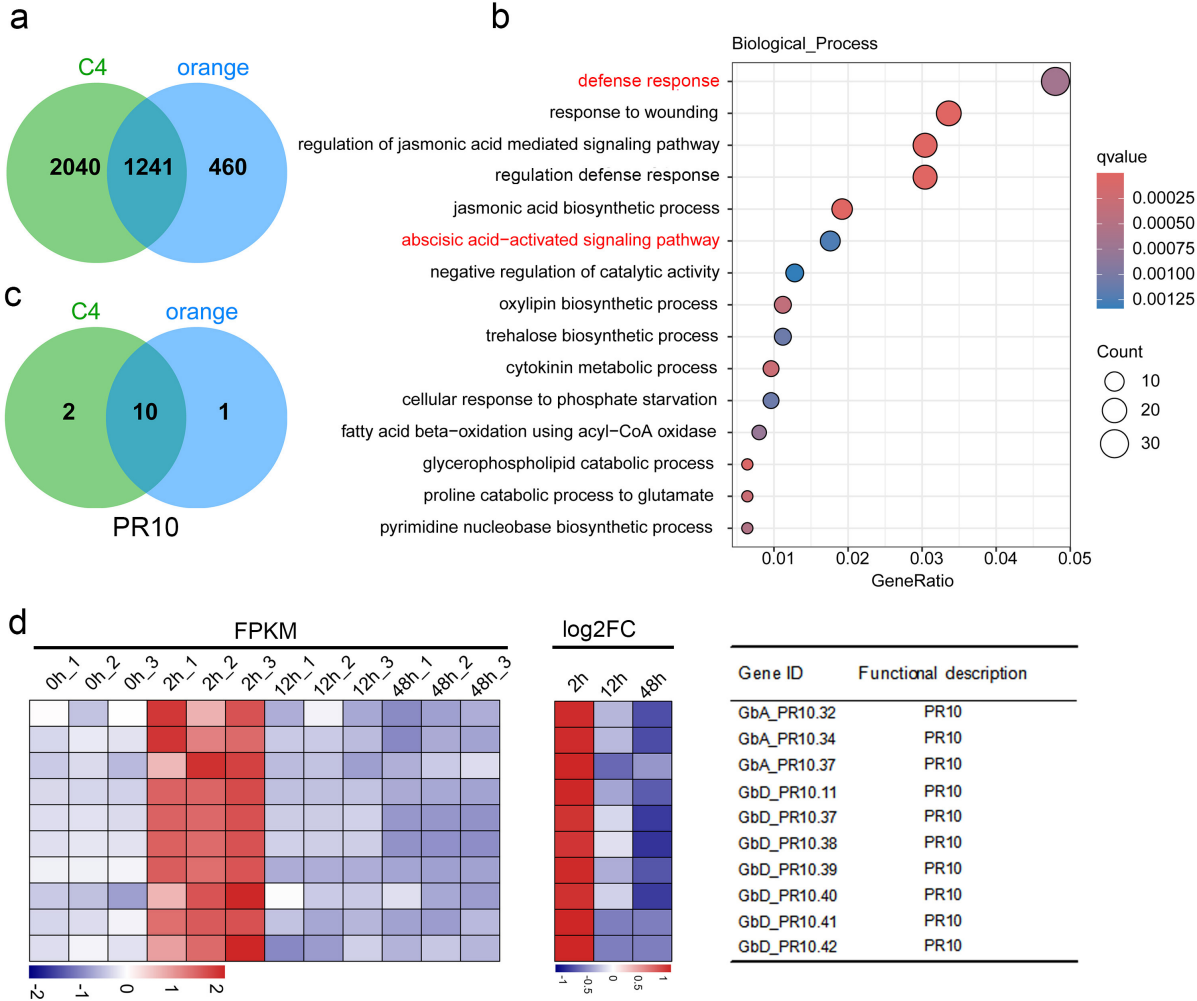
Integrate K-means clustering and WGCNA analysis. **(a)** Intersection of genes in cluster C4 (from K-means clustering) with those in the orange module (from WGCNA) identifies 1,241 shared genes. **(b)** Gene Ontology biological process enrichment analysis of these 1,241 shared genes. The pathways highlighted in red are enriched with the *PR10* genes. **(c)** Intersection of *PR10* genes in the enriched two pathways. **(d)** Expression pattern of the 10 shared genes in each sample. FC denotes the fold change between the control and different inoculation time.

By intersecting the early-response genes from Cluster C4 and the orange module, we identified a high-confidence set of 1,241 genes that represent the core of the immediate transcriptional defense (**Figure 4a**). GO analysis of these shared genes was significantly enriched in defense response and abscisic acid-activated signaling pathway (**Figure 4b**; **Supplementary Table S8**). Upon closer examination of the gene distribution within these pathways, we found that 10 out of 50 genes belong to the *PR10* gene family, including *GbA_PR10.32*, *GbA_PR10.34*, *GbA_PR10.37*, *GbD_PR10.11*, *GbD_PR10.37*, *GbD_PR10.38*, *GbD_PR10.39*, *GbD_PR10.40*, *GbD_PR10.41* and *GbD_PR10.42*. All genes showed strong and rapid induction at 2 hpi, marking them as key players in the initial defense against FOV7 (**Figures 4c, d**; **Supplementary Table S9**).

### 3.3 Evolutionary analysis reveals tandem duplication as the engine of *PR10* expansion

**Figure 5:**
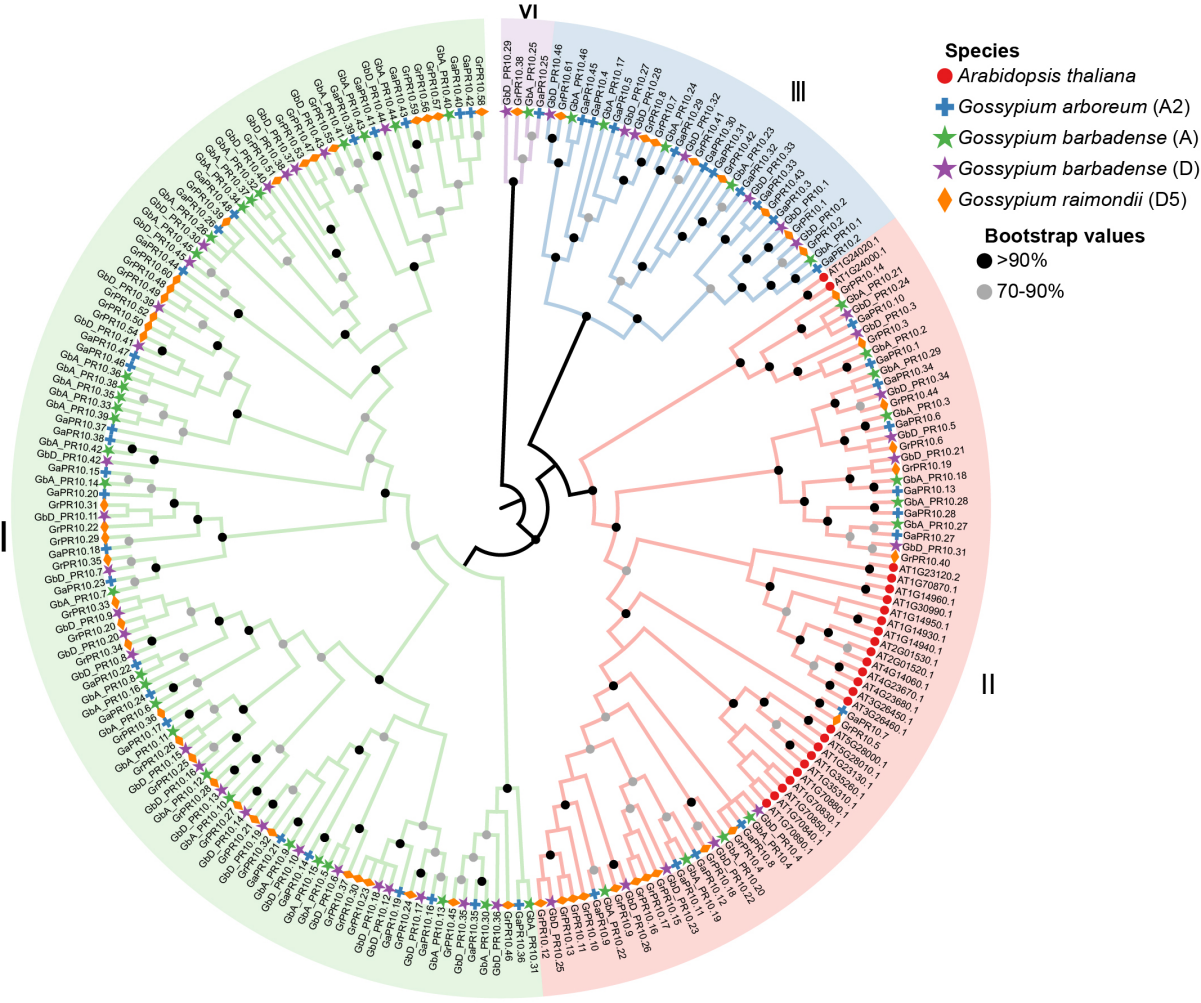
Phylogenetic ML tree of *PR10* genes from the three cottons and *A. thaliana*.

Having established the functional importance of *PR10* genes in the early defense response, we next investigated the evolutionary origins of this family in cotton. We performed a genome-wide identification and found 92, 48, and 61 *PR10* genes in *G. barbadense* (Gb), *Gossypium arboreum* (A2), and *Gossypium raimondii* (D5), respectively (**Supplementary Table S9**). A phylogenetic analysis including 26 *PR10* genes from *Arabidopsis thaliana* revealed four major clades, including Clades I, II, III, and IV (**Figure 5**). Among them, Clade I has the most, with a total of 116 members, Clade II has 77 members, Clade III has 30 members, and Clade IV has the fewest members with only four cotton members (*GbA_PR10.25*, *GaPR10.25*, *GbD_PR10.29*, *GrPR10.38*). Notably, *PR10* genes from all three cotton species are distributed across the four clades, while the *AtPR10* genes from *A. thaliana* are clustered exclusively in Clade II. This indicates that Clades I, III, and IV may be unique to cotton.

**Figure 6:**
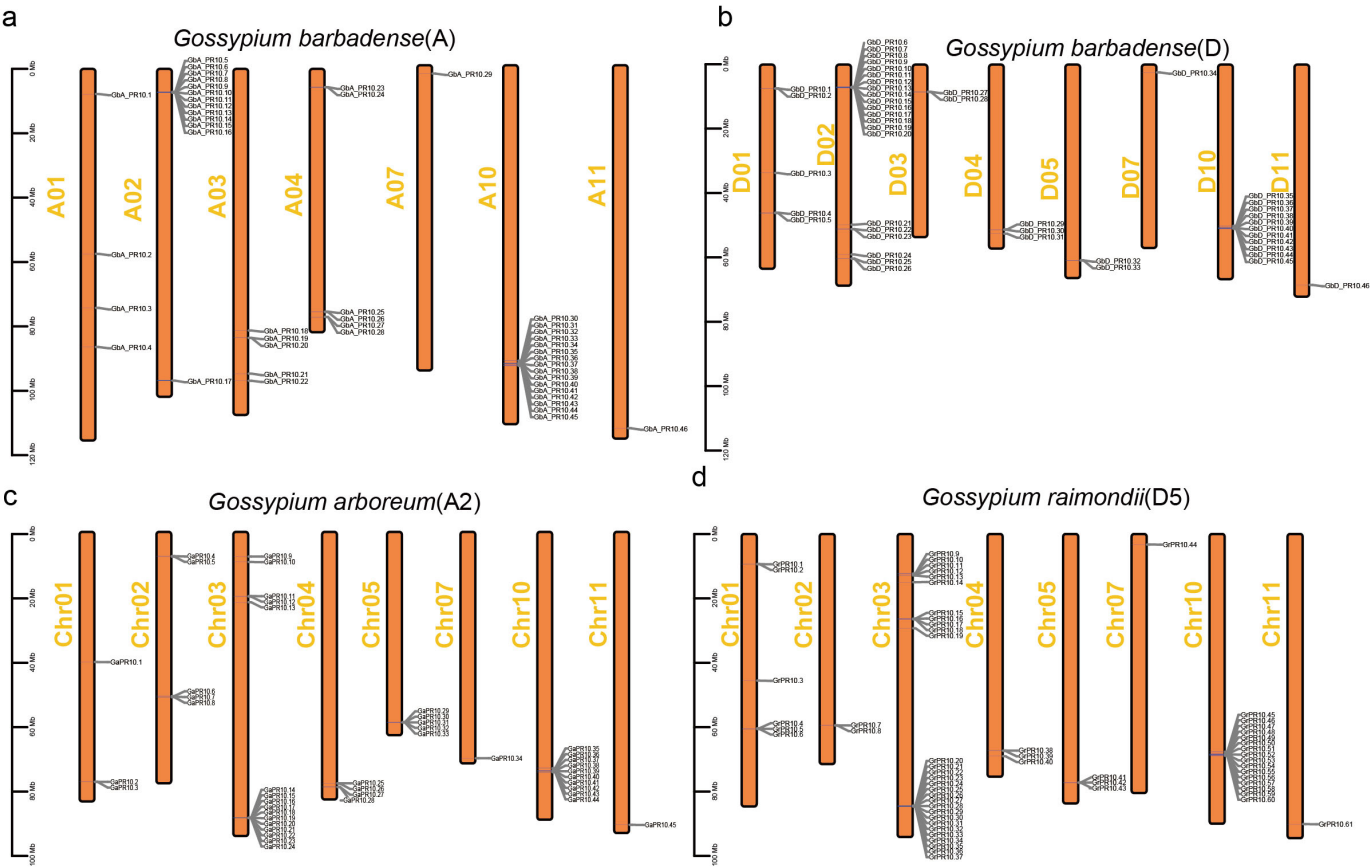
Chromosomal localization of *PR10* gene family in three cotton species. **(a)** *G*. *barbadense* A sub-genome, **(b)** *G*. *barbadense* D sub-genome, **(c)** *G*. *arboreum*, **(d)** *G*. *raimondii*.

**Figure 7:**
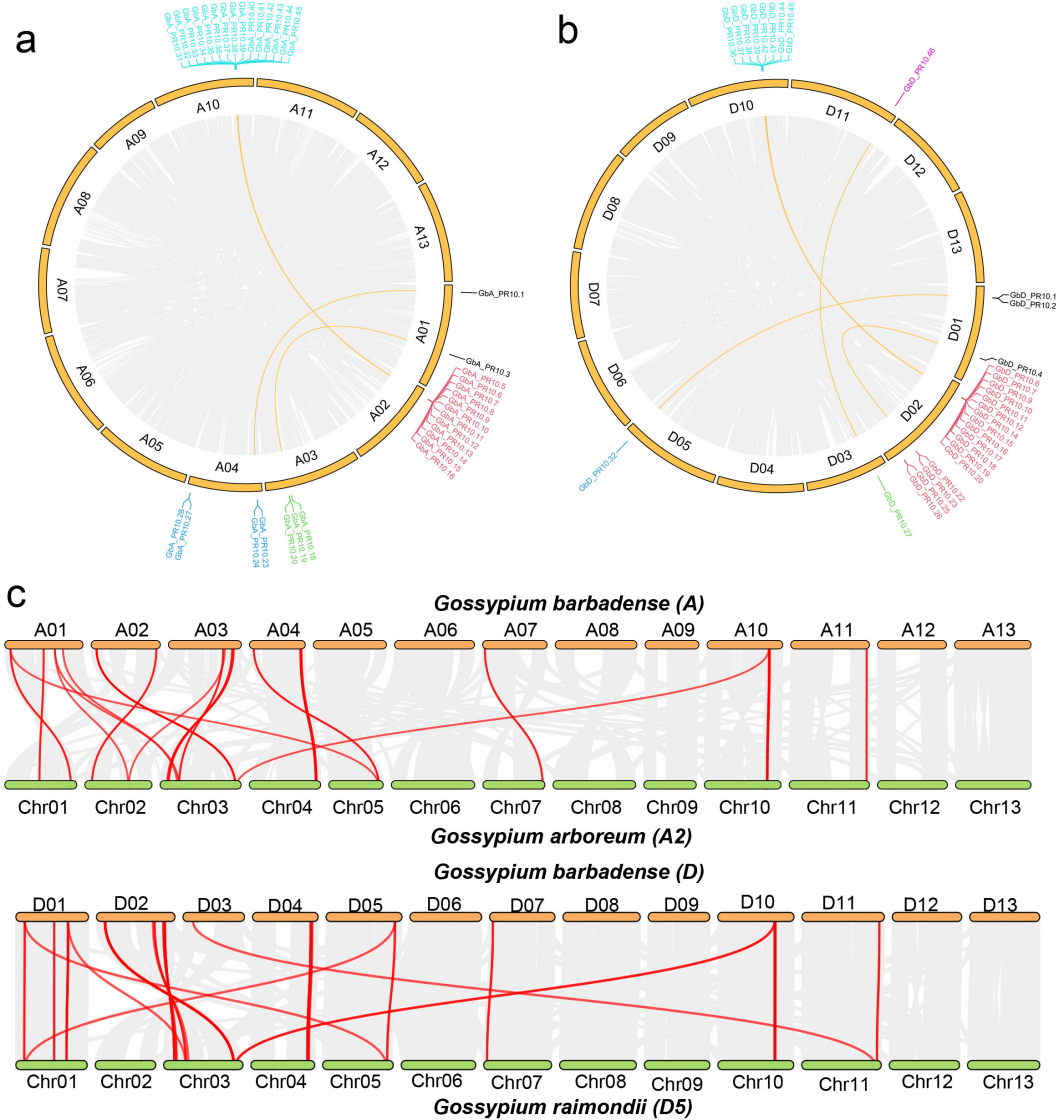
Synteny and collinearity analysis of cotton *PR10* gene family. **(a)** Circos plot of *PR10* genes in the *G*. *barbadense* A sub-genome **(a)** and the *G*. *barbadense* D sub-genome **(b)**. Segmental duplications of *PR10* gene pairs are linked and highlighted in yellow, and tandem *PR10* genes are also labeled. **(c)** Collinearity plot of *PR10* genes between the *G*. *barbadense* A sub-genome and *G*. *arboreum*, and between the *G*. *barbadense* D sub-genome and *G*. *raimondii*. Gray lines in the background indicate collinear blocks between genomes, while red lines denote syntenic *PR10* gene pairs.

Chromosomal mapping in *G. barbadense* showed a highly non-uniform distribution of the 92 *Gb_PR10* genes. We identified four major gene clusters located at the ends of chromosomes A02, A10, D02, and D10, which together accounted for 58.7% of all *Gb_PR10* genes (**Figure 6**). Gene synteny and collinearity analysis revealed that tandem duplication was the primary driving force behind the expansion of these clusters, with 56.5% of genes in the A sub-genome and 39.1% in the D sub-genome being tandem duplicates (**Figures 7a, b**; **Supplementary Table S9**; **Supplementary Figure S2**). The identification of large syntenic blocks between chromosomes A02/A10 and D02/D10 suggests these clusters arose from an ancient whole-genome duplication (WGD) event, a known feature of the *Gossypium* lineage (Paterson et al., 2012). Furthermore, Ka/Ks analysis of these tandem pairs indicated that they are under strong purifying selection (Ka/Ks < 1), suggesting their functional importance (**Supplementary Table S10**).

### 3.4 The tandemly duplicated *PR10* cluster on chromosome D10 is a hub for the FOV7 response

**Figure 8:**
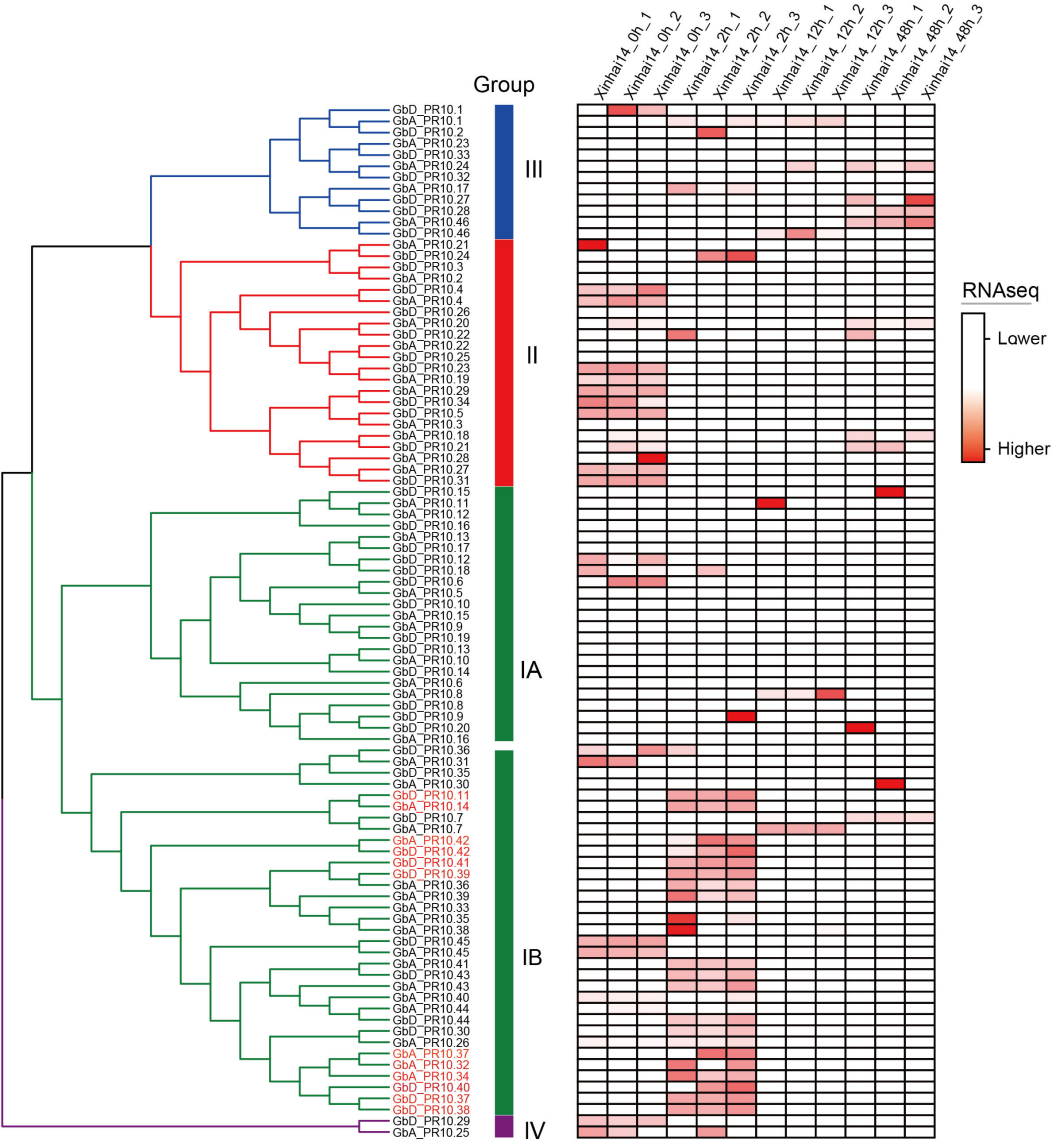
Expression patterns of *Gb_PR10* gene family at different time during FOV7 infection of cotton. The gene expressions were made row -scaled. The genes marked in red are those with significantly upregulated at 2 hpi.

We then integrated our evolutionary and expression analyses to ask whether these expanded gene clusters were linked to the defense response. By mapping the expression data of all 92 *Gb_PR10* genes, we made a striking observation: the genes that were significantly upregulated at 2 hpi were overwhelmingly located within the tandem gene cluster on chromosome D10 (**Figure 8**; **Supplementary Table S11**). Twelve of the thirteen significantly upregulated genes belonged to this cluster, including the highly expressed *GbD_PR10.38*, *GbD_PR10.11*, and *GbD_PR10.37*. This directly links the evolutionary expansion of a specific genomic region to a critical functional role in pathogen defense.

### 3.5 Single-nucleus transcriptomics localizes the early defense response to the root epidermis

**Figure 9:**
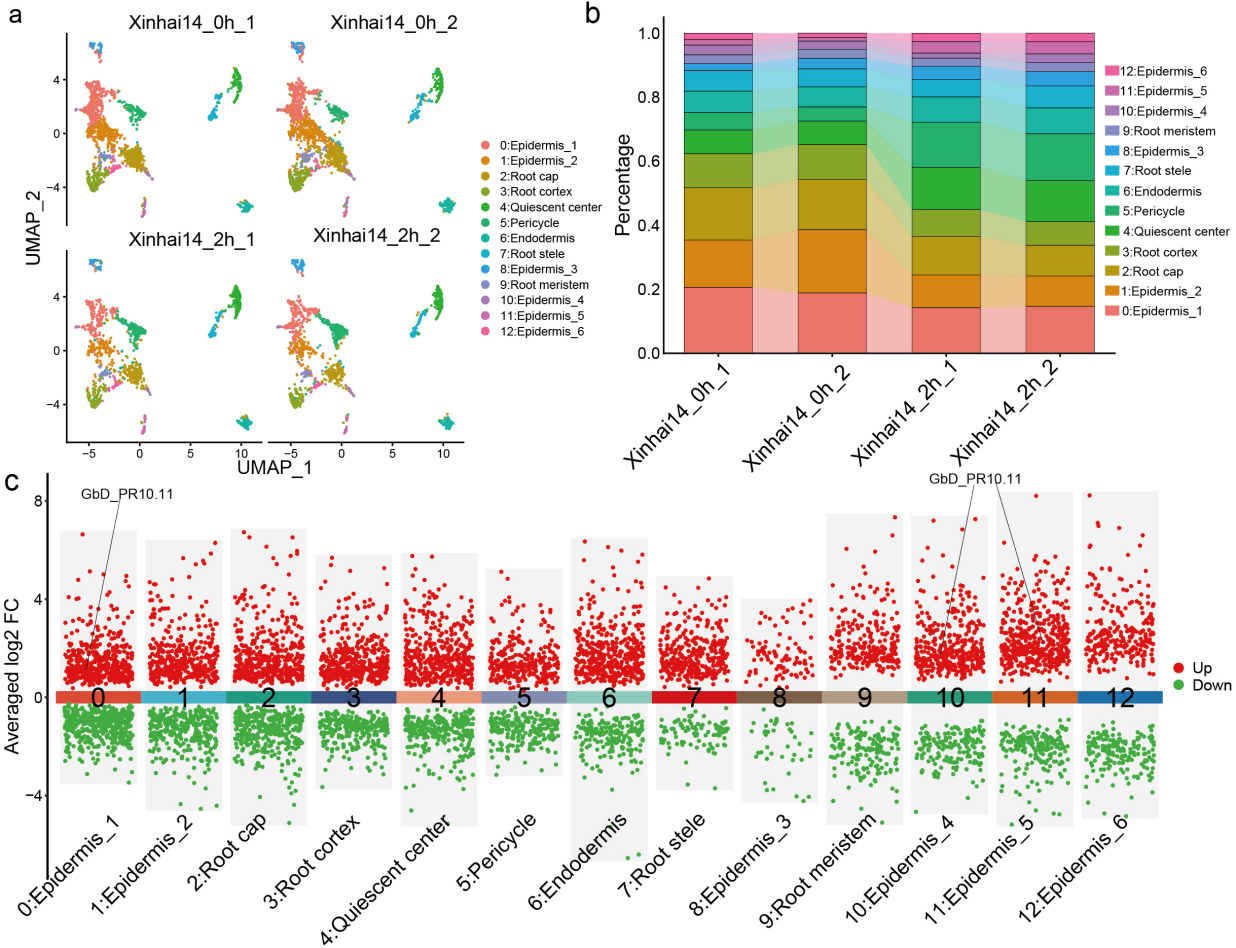
Single cell transcriptome analysis of *G*. *barbadense* root cells during FOV7 infection. **(a)** UMAP dimensionality reduction plot showing different cell types. The figure includes four distinct samples: two samples of Xinhai14 (Xinhai14_0h_1 and Xinhai14_0h_2) as controls and two samples of Xinhai14 at 2 hpi (Xinhai14_2h_1 and Xinhai14_2h_2) for treatment. Each dot represents a single cell, with different colors indicating different cell clusters. **(b)** Bar plot showing the percentage distribution of different cell types across the four samples. Each bar represents a sample, with different colors corresponding to different cell clusters. **(c)** Differential expression analysis across different cell clusters, with *PR10* genes directed in the plot.

While our bulk RNA-seq analysis identified the key defense genes, it could not resolve which cell types were responsible for this rapid response. To address this, we performed single-nucleus RNA-seq (snRNA-seq) on root tips from control and 2 hpi samples. After stringent quality control, we obtained a total of 7,843 high-quality cells, comprising 4,723 cells from the control group and 3,120 cells from the 2-hour treatment group. These cells were subsequently grouped into 13 distinct cell clusters (**Figure 9a**; **Supplementary Table S13**). Based on known marker gene expression (Denyer et al., 2019; Huang et al., 1996; Li et al., 2024; Shahan et al., 2022; Zhu et al., 2023), we successfully annotated various root cell types, with epidermal cells being the most abundant population (**Figure 9b**; **Supplementary Figures S3**; **S4**; **Supplementary Table S14**).

Upon comparing the transcriptomes of infected versus control samples at the cellular level, we observed cell-type-specific responses to FOV7 (**Figure 9c**; **Supplementary Table S15**). Critically, when we examined the expression of the *PR10* gene family, we found that *GbD_PR10.11*—one of the highly induced genes from the chromosome D10—was specifically and significantly upregulated in three epidermal sub-clusters (Clusters 0, 10, and 11) at 2 hpi. This finding pinpoints the epidermis as a primary defensive frontier and identifies *GbD_PR10.11* as a key sentinel gene activated in these frontline cells, demonstrating pronounced cellular heterogeneity in the immune response.

### 3.6 RT-qPCR confirms the expression patterns of key candidate *PR10* genes

**Figure 10:**
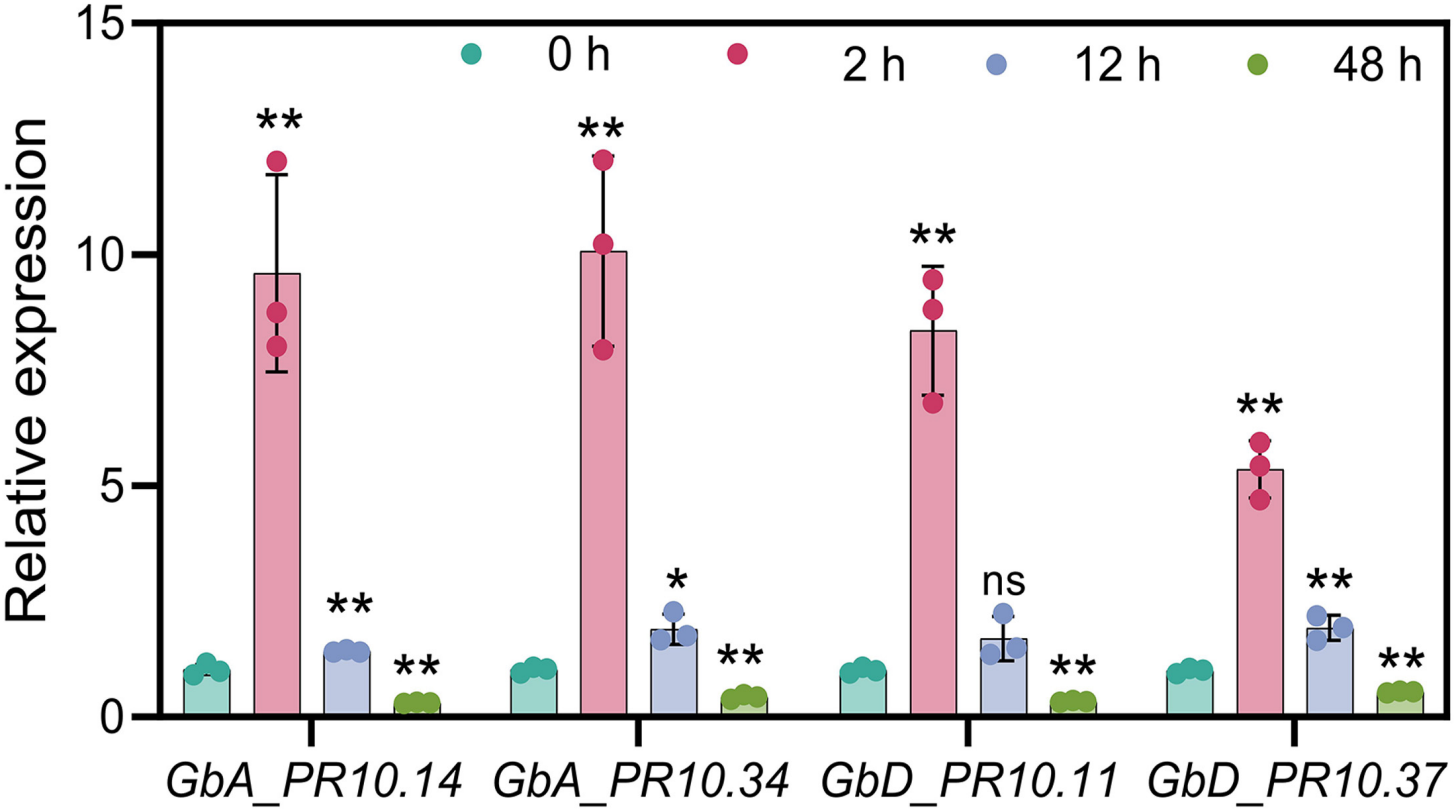
qRT-PCR results of four selected *GbPR10* genes under FOV7 infection stress. * represents p < 0.05, ** represents p < 0.01.

To validate our sequencing results, we selected four candidate *PR10* genes representing different aspects of our findings and performed RT-qPCR analysis. The selected genes included *GbA_PR10.34* (identified by both K-means and WGCNA), *GbD_PR10.11* (the key gene specifically expressed in the epidermis), and two other highly expressed genes from the tandem clusters (*GbA_PR10.14* and *GbD_PR10.37*). Consistent with our RNA-seq data, all four genes showed significant upregulation at 2 hpi, confirming their crucial involvement in the early defense response against FOV7 (**Figure 10**).

## 4 Discussion

Despite the substantial economic impact of FOV7 on cotton (Diaz et al., 2021), the intricacies of the defense mechanisms for FOV7 infection remain largely unexplored. In this study, we dissected the early host-pathogen arms race between *G. barbadense* and FOV7 at a multi-scale resolution. By integrating transcriptomics from bulk tissue down to the single nucleus, we moved beyond a simple catalog of defense genes to uncover an elegant defense strategy. Our findings connect a specific evolutionary mechanism—tandem duplication of *PR10* genes—to a functional genomic hotspot on chromosome D10, and ultimately pinpoint the root epidermis as the primary cellular battleground where a key sentinel gene, *GbD_PR10.11*, is deployed. This research successfully demystifies the intricate, cell-specific immune responses that occur at the very onset in cotton roots. Further evolutionary analysis *PR10* genes in cottons revealed that the chromosome D10 acts as a genomic hotspot for adaptation (**Figures 5**–**7**). The concentration of responsive *PR10* genes in this tandem cluster (**Figure 8**) suggests it may function as a co-regulated genomic cassette, where shared regulatory elements could enable a swift, synchronized, and high-magnitude transcriptional response upon pathogen recognition. This architecture provides a potent gene dosage effect, which is a classic evolutionary strategy to counter pathogen pressure (Khanfir et al., 2024; Xu et al., 2024).The multiple gene copies within this cluster may not be merely redundant. It is plausible that they have undergone sub- or neofunctionalization, evolving slightly different expression patterns, enzymatic activities, or affinities for pathogen effectors, thus providing a more versatile and robust “defensive toolkit”. From a breeding perspective, such a functionally significant gene cluster represents a prime target for marker-assisted selection or even for transfer as a complete unit to enhance resistance in susceptible elite varieties (Chen LiJun et al., 2013).

A key breakthrough of our study is the precise localization of this early defense to the epidermis. This finding positions the root epidermis not as a passive barrier, but as an active immune frontier (**Figure 9**). As the first point of contact, a rapid defense in this layer is strategically critical (Ali et al., 2024; Chen LiJun et al., 2013; Sidiq et al., 2022). We speculate that *GbD_PR10.11* acts as a first responder. Its molecular function could be multifaceted: its RNase activity might directly degrade pathogen-derived RNA molecules, or upon programmed cell death, the protein could be released into the apoplastic space to attack the fungus directly (Dos Santos and Franco, 2023; Karimian et al., 2024). Alternatively, its ligand-binding pocket could sequester pathogen effectors or bind endogenous signaling molecules to modulate the defense response (Khan et al., 2025; Przybylska and Obrępalska-Stęplowska, 2020). This highlights a clear cellular division of labor, where the epidermis mounts a rapid, direct defense, likely while the underlying cortical and vascular tissues initiate longer-term responses like cell wall reinforcement and systemic signaling.

While our study provides a high-resolution snapshot of the early infection events, we acknowledge its limitations. Our analysis focused on the initial 48 hours and a single cotton cultivar. The roles of these *PR10* genes in later infection stages and across different genetic backgrounds warrant further investigation. Moreover, while snRNA-seq provides invaluable spatial information, the functions of the identified genes require direct validation. Future research should therefore prioritize the functional characterization of *GbD_PR10.11* and other promising candidates from the D10 cluster using gene editing (e.g., CRISPR-Cas9) and overexpression systems. Such studies will be crucial to confirm their roles in FOV7 resistance and to elucidate their underlying molecular mechanisms, be it through RNase activity, ligand binding, or other functions.

## 5 Conclusions

This research bridges genomics, cell biology, and pathology to paint a cohesive picture of cotton’s frontline defense. We have identified a tandemly-amplified *PR10* gene cluster that is functionally deployed in the epidermis. The sentinel gene *GbD_PR10.11* represents a high-value target for a new generation of “precision breeding”. Instead of targeting genes with broad, constitutive expression that may incur a fitness cost, our work paves the way for engineering a fortified cellular barrier—enhancing frontline immunity in the exact cells where it is needed most. This strategy holds the promise of developing a more sophisticated and durable resistance against one of cotton’s most formidable pathogens.

## Data Availability

The datasets presented in this study can be found in online repositories. The names of the repository/repositories and accession number(s) can be found in the article/**Supplementary Material**.
