# Supplementary material for: Integrated RNA-seq and snRNA-seq analysis identifies PR10 tandem gene cluster governing early defense against Fusarium wilt in sea island cotton
Source: Front Plant Sci. 2025 Nov 5;16:1622223. doi: 10.3389/fpls.2025.1622223 (PMC12627019; doi:10.3389/fpls.2025.1622223)
Supplement: Supplementary file 1 [file DataSheet1.docx]

**Supplementary Figures**

**
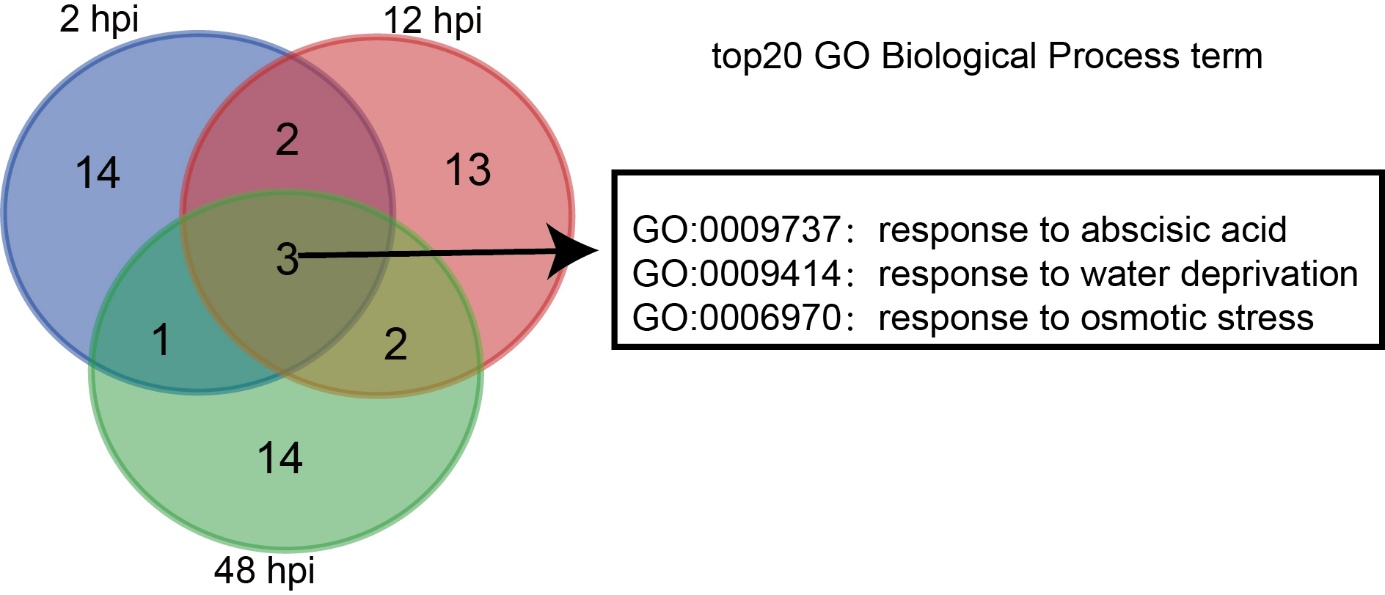
**

**Figure S1.** Venn diagram to visually emphasize the common core early response pathway across time points


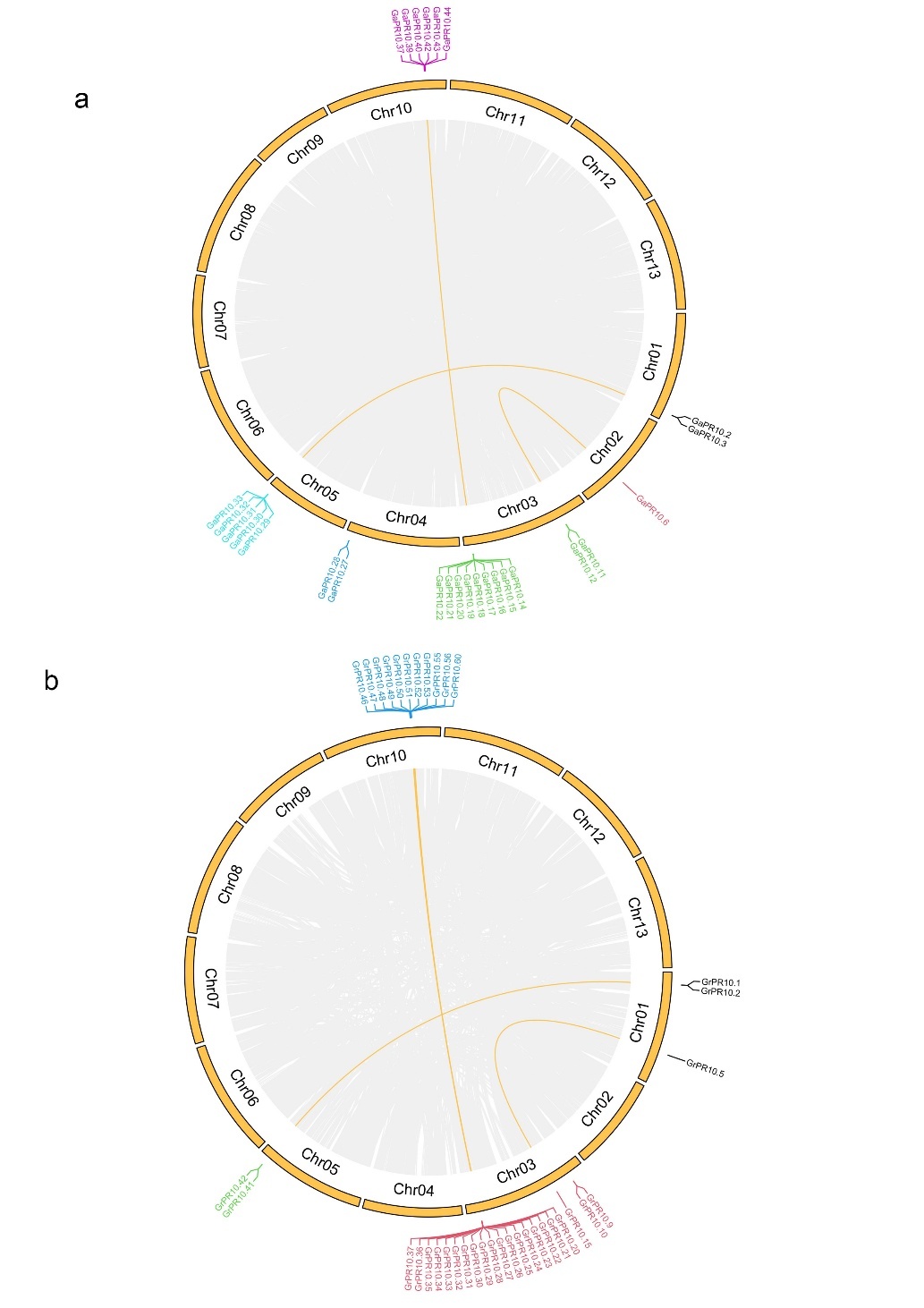


**Figure S2.** Circos plot of *PR10* genes in the *G. arboreum* (a) and *G. raimondii* (b)*.*


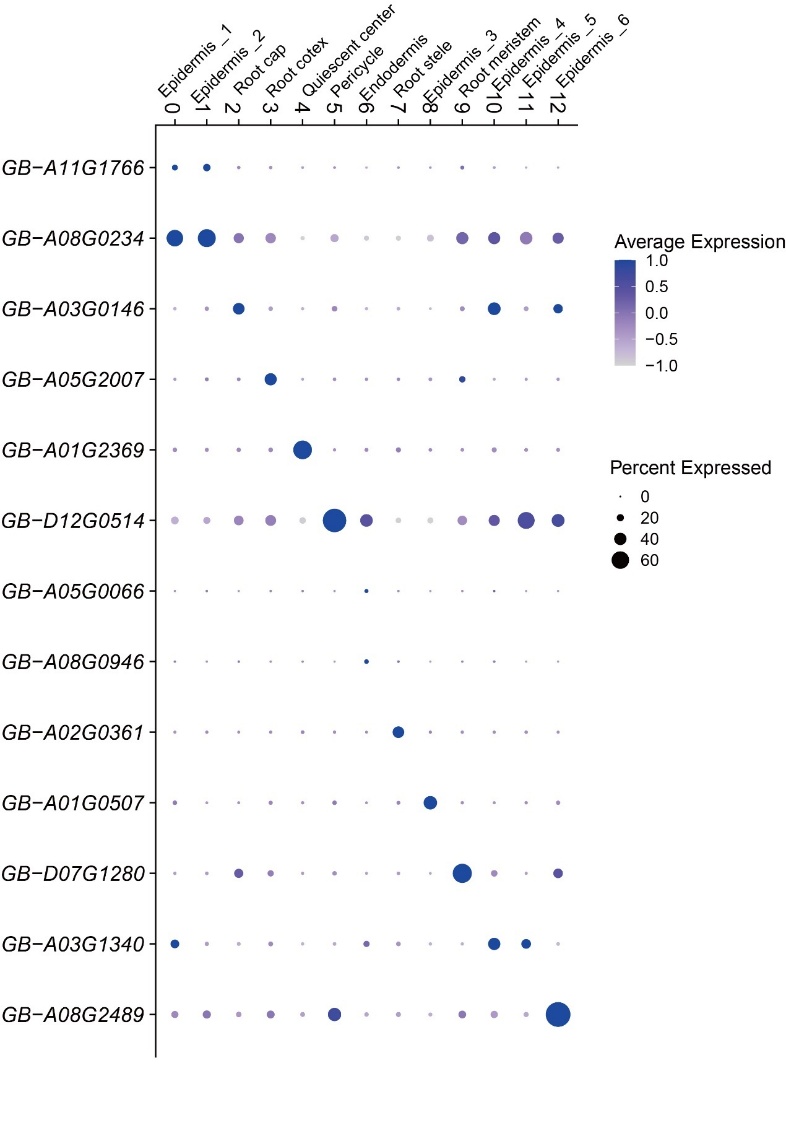


**Figure S3.** Dotplot selected marker genes.


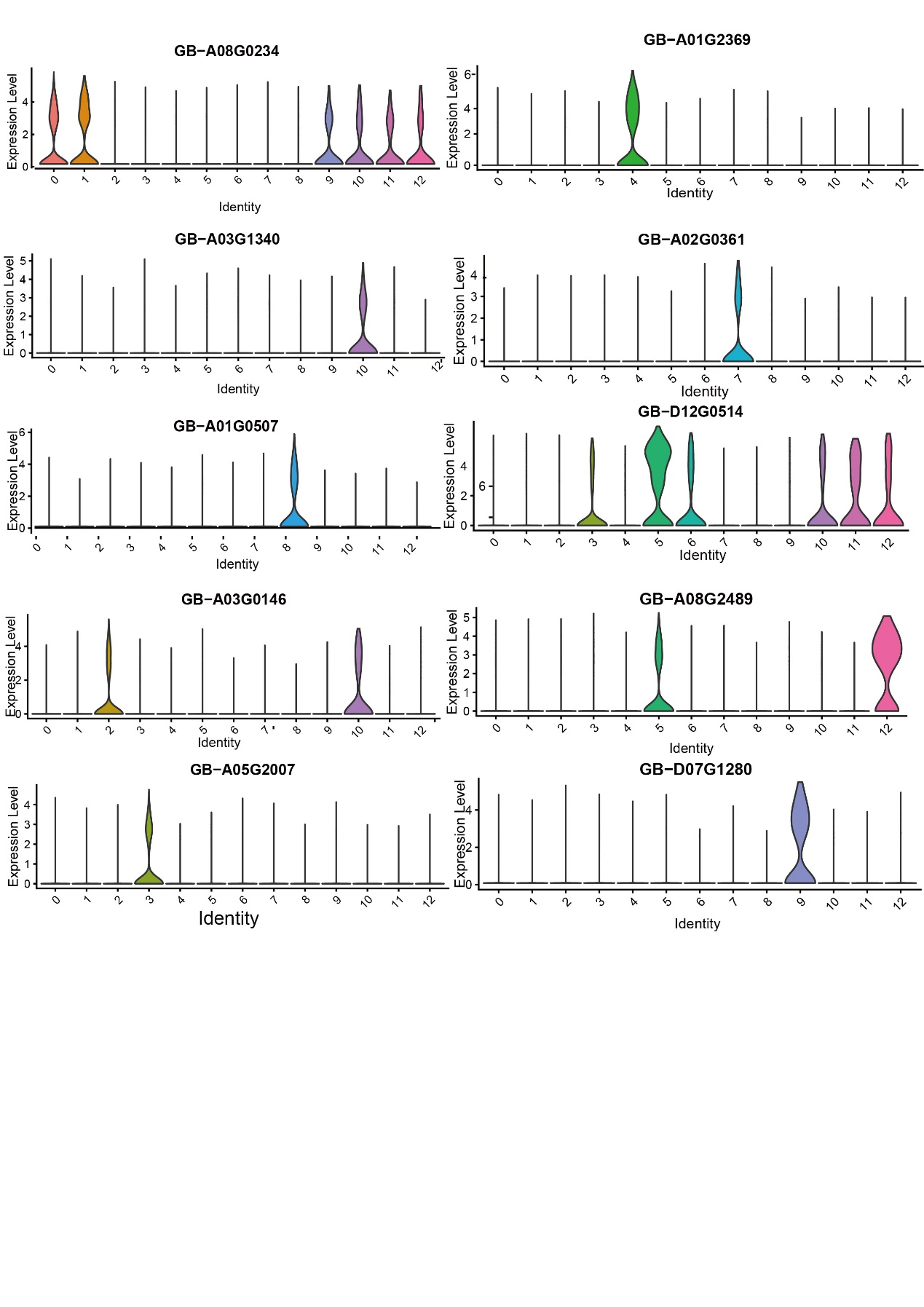


**Figure S4.** Violin plot of selected marker genes*.*
